# The relationship between locomotion and hindlimb morphology in the leopard (*Panthera pardus*) using a geometric morphometric approach

**DOI:** 10.1242/bio.061823

**Published:** 2024-12-24

**Authors:** Riyanta Naidoo, Safiyyah Iqbal

**Affiliations:** School of Animal, Plant and Environmental Sciences, University of the Witwatersrand, Wits 2050, Johannesburg, Gauteng 2000, South Africa

**Keywords:** Behaviour, Geometric morphometrics, Hindlimb morphology, Locomotion, Niche preference

## Abstract

Felid bone morphology is highly influenced by factors such as locomotion, body size, and foraging behaviour. Understanding how these factors influence bone morphology is important for interpreting the behaviour and ecology of such species. This study aimed to determine the extent to which *Panthera pardus* (i.e. leopard) hindlimb morphology differs from that of other *Panthera* species, particularly *Panthera leo* (i.e. lion). Landmark-based geometric morphometric analyses were used to compare 27 *Panthera* femurs in the anterior and posterior views, by the use of principal component analyses. Distinct clusters were found linking the *Panthera* species for both the anterior and posterior views, inferring a difference in the femur morphology of the species. The Procrustes ANOVA regression further showed a significant difference in the mean shape between the *Panthera* femurs, for both the anterior and posterior views. A clear relationship was found between femur morphology and body size, with leopards possessing a more gracile and elongated femur to support a smaller body mass and lions possessing a more robust and stunted femur to support a larger body mass. It was found that femur morphology also correlates with locomotive flexibility and hunting success in felids. Leopard femur morphology aids in speed and flexibility during hunting, as well as aids in propulsion that allows for arboreal locomotion. It was ultimately deduced that femur morphology differs between *Panthera* species, according to their mechanical demands during locomotion.

## Teaser

**Summary:** This study highlights significant differences in femur morphology between *Panthera* species, linking these variations to body size, locomotion, and ecological adaptations like hunting success and arboreal movement.

## INTRODUCTION

Locomotor modes may differ due to body size, the need for speed, or movement over longer distances (Alvarez et al., 2013). These factors contribute to certain needs on the axial skeleton and particular demands on the appendicular skeleton and respective musculature (Alvarez et al., 2013). Locomotion brings about mechanical stress on the postcranial skeleton in mammals, thereby having effects on the bone morphology and the organization of muscles attached to the bone (Alvarez et al., 2013). Consequently, these bones need to be well adapted to withstand these stresses. An increase in body size is correlated with an increase in mechanical stress (Biewener, 1990). Therefore, larger mammals accommodate for this increased stress by supporting their body mass with a more straight and rigid vertebral column, with their limbs situated directly under their torso to increase stability during locomotion (Biewener, 1990; Fabre et al., 2013b). A mammal's limb posture also aids in supporting their body mass during locomotion (Day and Jayne, 2007). This is due the fact that it influences movement patterns as well as muscular activity, thereby accommodating for the bone stresses that come with changes in body size (Day and Jayne, 2007; Zhang et al., 2012). Larger mammals primarily have an upright posture that sustains mechanical stresses by having an increased mechanical advantage; therefore, these larger mammals have more robust limbs that decrease their locomotor performance (Biewener, 1990). Accordingly, smaller mammals have a more crouched posture with weaker limbs that bring about skeletal stiffness, as these smaller species undergo decreased amounts of bone stresses (Biewener, 1990, 1989; Bertram and Biewener, 1990).

Felids are extremely diverse in their locomotion as their locomotor modes are highly correlated with the various habitats that they exploit, including forests, grasslands, savannas, as well as deserts (Gonyea, 1976; Dev et al., 2020). Felid locomotor modes vary from terrestrial locomotion to scansorial and arboreal locomotion (Walmsley et al., 2012). Arboreal environments are more complex, with increased variation in the substrate; therefore, there is an increase in the selective pressures on the bone morphology for felids to successfully adapt to differing locomotor modes (Granatosky, 2018). For example, in rainforests, there is high variation in substrates consisting of dense vegetation, trees, and vines; therefore, felids occupying these habitats (such as leopards, jaguars and tigers) need to have more diverse locomotor behaviours. On the other hand, terrestrial environments (such as savannas and grasslands) are much less complex (Granatosky, 2018). These environments have more homogeneous substrates; therefore, felids occupying these habitats (such as servals, wild cats and lions) would not need such diverse locomotor modes (Granatosky, 2018).

Felid bone morphology is highly influenced by different factors such as locomotion and foraging behaviour; therefore, their skeletal structure needs to fulfil the requirements needed to sustain these factors with a minimum cost to the animal (Doube et al., 2009). The felid postcranial skeleton has various adaptations to different traits linked to locomotion, posture, and the hunting of prey (Walmsley et al., 2012). Most felids tend to stalk their prey by crouching when hunting, and pouncing on them when they are within striking distance (Kitchener et al., 2010). Many felids also have the ability to hunt arboreally, ambush terrestrial prey from trees, or even hunt for prey in water (Kitchener et al., 2010). Felids that occupy arboreal environments have a more slender postcranial skeleton with specialized morphological adaptations and a reduced body mass. This increases locomotor agility, as well as speed and precision when hunting for prey (Alvarez et al., 2013; Christiansen, 2002; Chen and Wilson, 2015). Arboreal felids have much longer limb output levers with a more slender humerus, a small and round humeral head, a slender femur, and a tibia with a reduced greater trochanter and an elongated shaft, all of which add to speed and agility adaptations (Chen and Wilson, 2015; Meachen-Samuels and Van Valkenburgh, 2009). Conversely, felids that are primarily terrestrial have a more robust postcranial skeleton with limb output levers that are much shorter (Chen and Wilson, 2015). These terrestrial felids are more adapted for powerful leaps as well as rapid acceleration over short distances (Kitchener et al., 2010; Meachen-Samuels and Van Valkenburgh, 2009). This is due to them having a more robust humerus and a larger humeral head, as well as a thicker and shorter femur shaft with a moderately built greater trochanter (Kitchener et al., 2010; Chen and Wilson, 2015; Meachen-Samuels and Van Valkenburgh, 2009).

This study primarily focuses on *Panthera pardus*, commonly known as the leopard. The leopard is the smallest species of ‘big cats’ that falls under the *Panthera* genus, which also includes lions, tigers, and jaguars (Stein and Hayssen, 2013; Jacobson et al., 2016). Leopards can be found in a wide range across Africa and Asia, from the Middle East to the Pacific Ocean (Jacobson et al., 2016). Their behavioural plasticity allows them to live in almost any type of habitat due to their ability to easily adapt to changing environmental conditions (Jacobson et al., 2016). Leopards are the most arboreal of the *Panthera* genus as they are known to chase or haul their prey into trees when hunting, as well as to retreat to trees when they feel threatened (Hunter et al., 2013). They are strong climbers and adept swimmers that often hunt in water (Hayward et al., 2006). They have long slender bodies with shorter limbs that aid in arboreal locomotion as they are well adapted for grasping or holding onto trees (Kitchener et al., 2010). Further, they have long tails that assist with balance when pacing on tree branches (Kitchener et al., 2010). Leopard limbs are well equipped with an increase in musculature that aids in hunting strategies (Kelly and Sears, 2011). Their forelimbs are more adapted for grasping and pouncing on prey and the hindlimbs for propulsion (Kelly and Sears, 2011; Fabre et al., 2013a). This increased musculature results in an increased load on weight-bearing bones in the limbs (i.e. the femur in the hindlimbs) (Biewener, 1989); therefore, the morphology of these weight-bearing bones needs to be accommodating to the muscle attachment in order to help with movement and stability during locomotion.

Most studies focus on the forelimbs in mammals and how they are adapted for various modes of locomotion and hunting (Fabre et al., 2013b; Zhang et al., 2012; Walmsley et al., 2012; Andersson, 2004; Martin-Serra et al., 2014); therefore, there is a lack of knowledge on the hindlimb morphology in felids and the inferences that can be made from femurs regarding locomotion. This study will give insight to better our understanding of how locomotion, alongside foraging behaviour and prey capture, contributes to the bone morphology in the hindlimbs of the *Panthera* species. Understanding how bone morphology is influenced by factors such as locomotion is important for interpreting the behaviour and ecology of this species, and provides a quantitative outline for the analysis of other species.

The leopard is classified as vulnerable by the International Union for the Conservation of Nature (IUCN) (Jacobson et al., 2016). Illegal trade and poaching continue to threaten the survival of the species as leopard or other felid bones are used for traditional medicinal practices (Podhade et al., 2013). There is a belief that these bones bring physical strength and good health to consumers (Podhade et al., 2013). This study could help to raise awareness of conservation as well as promote rehabilitation of the leopard species. Further, it will assist in optimizing diagnosis and treatments. For example, if an animal is kept in conservation or in rehabilitation, one would be able to improve on the diagnosis for disorders. This study could also aid in detecting abnormalities in the motor system or musculo-skeletal structure in mammals.

The aim of this study was to determine the extent to which the *Panthera pardus* hindlimb morphology differs from that of other *Panthera* species. The objectives were to: (1) make a comparative assessment of the *Panthera* hindlimb morphology, (2) determine whether there are any distinct morphological clusters on the hindlimb that may link the different *Panthera* species, and (3) determine the impact that femur morphology has on *Panthera* locomotion.

## RESULTS

**Fig. 1.**
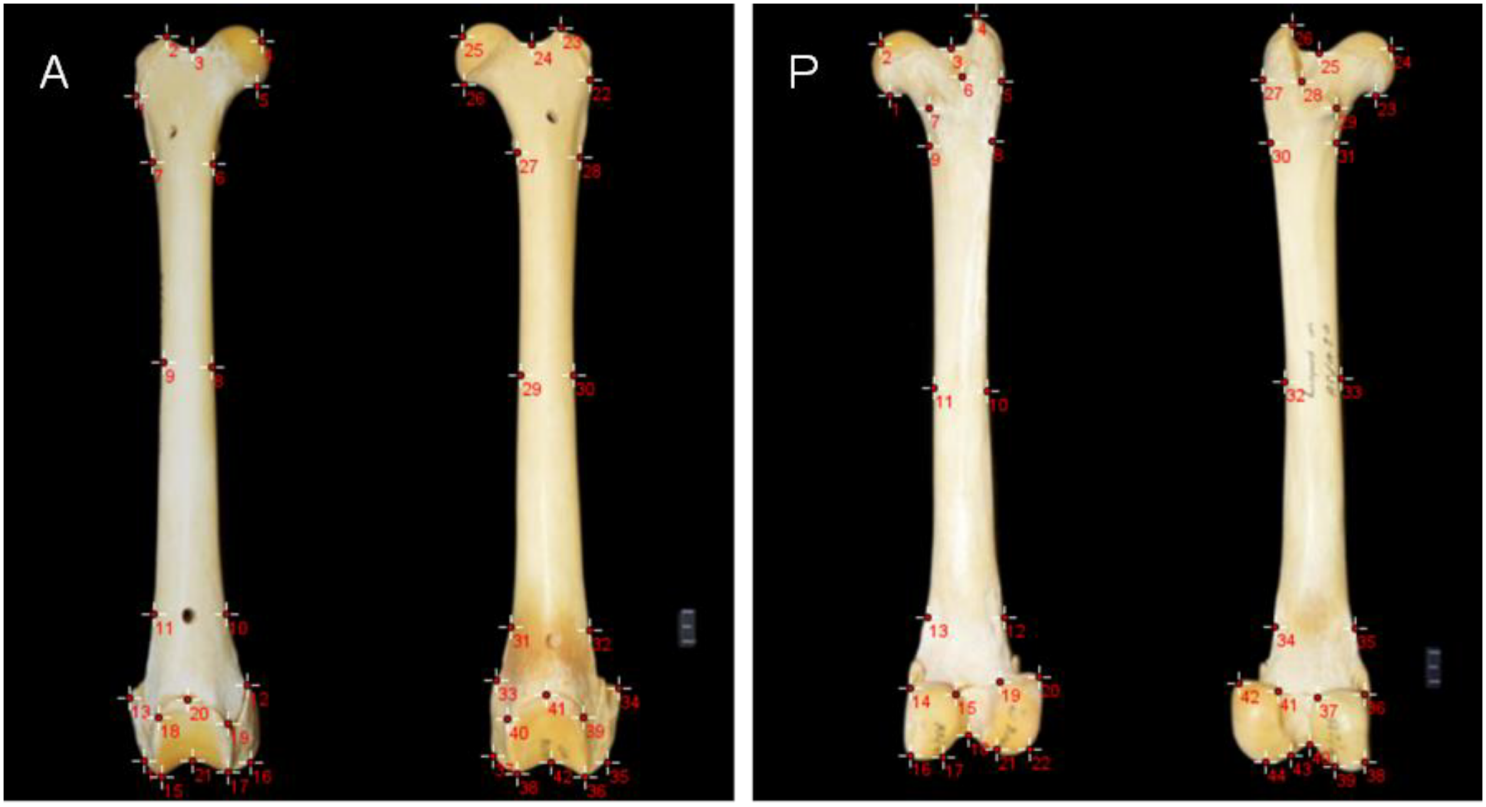
**Positioning of landmarks on the anterior and posterior views of the *Panthera* femurs.** A, anterior; P, posterior. Landmarks are indicated by red points on the femurs.

**Fig. 2.**
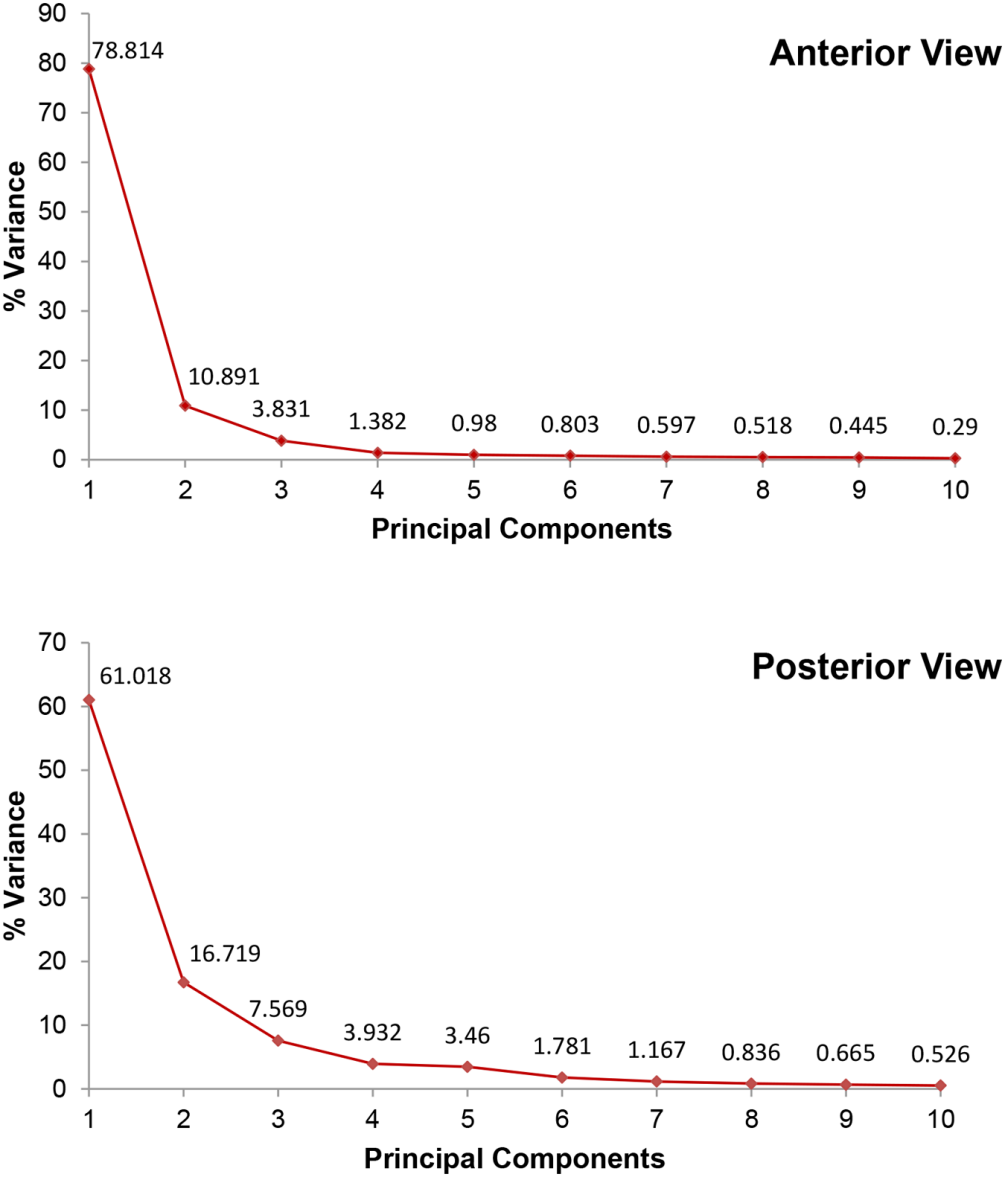
Scree plot representing the percentage variance (eigenvalues) of the first ten principal components for the anterior and posterior views of the *Panthera* femurs.

**Fig. 3.**
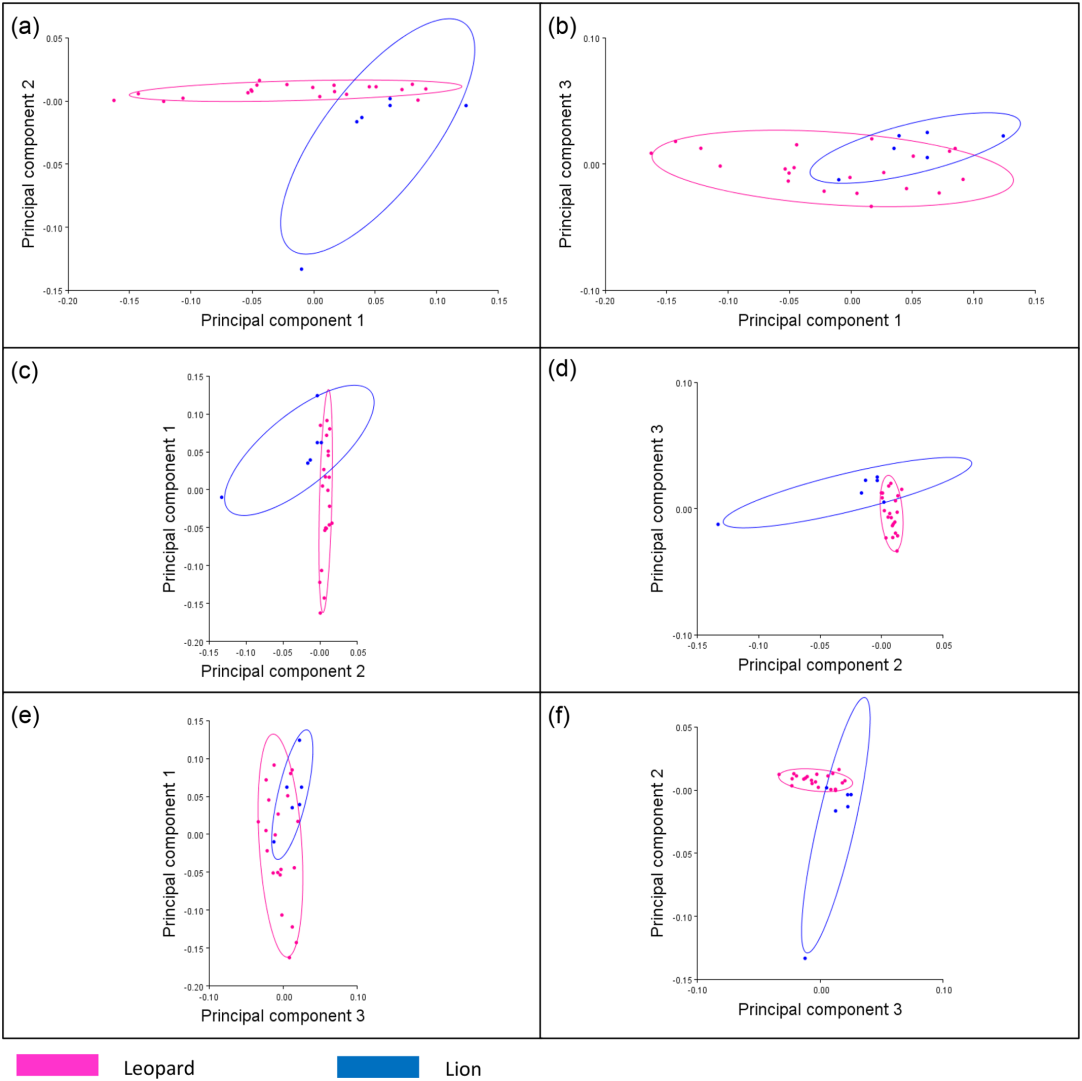
**Principal component analysis (PCA) plots showing the relationships between the first three principal components for the anterior view of the *Panthera* femurs.** Leopard femurs are represented in pink and lion femurs are represented in blue. Clusters for both the leopard and lion femurs are indicated by confidence ellipses with their respective colouration.

**Fig. 4.**
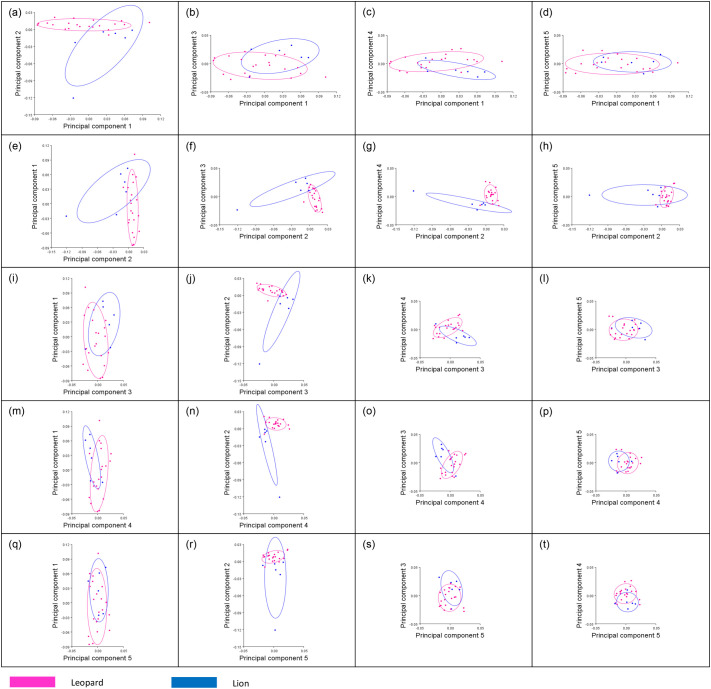
**PCA plots showing the relationships between the first five principal components for the posterior view of the *Panthera* femurs.** Leopard femurs are represented in pink and lion femurs are represented in blue. Clusters for both the leopard and lion femurs are indicated by confidence ellipses with their respective colouration.

**Fig. 5.**
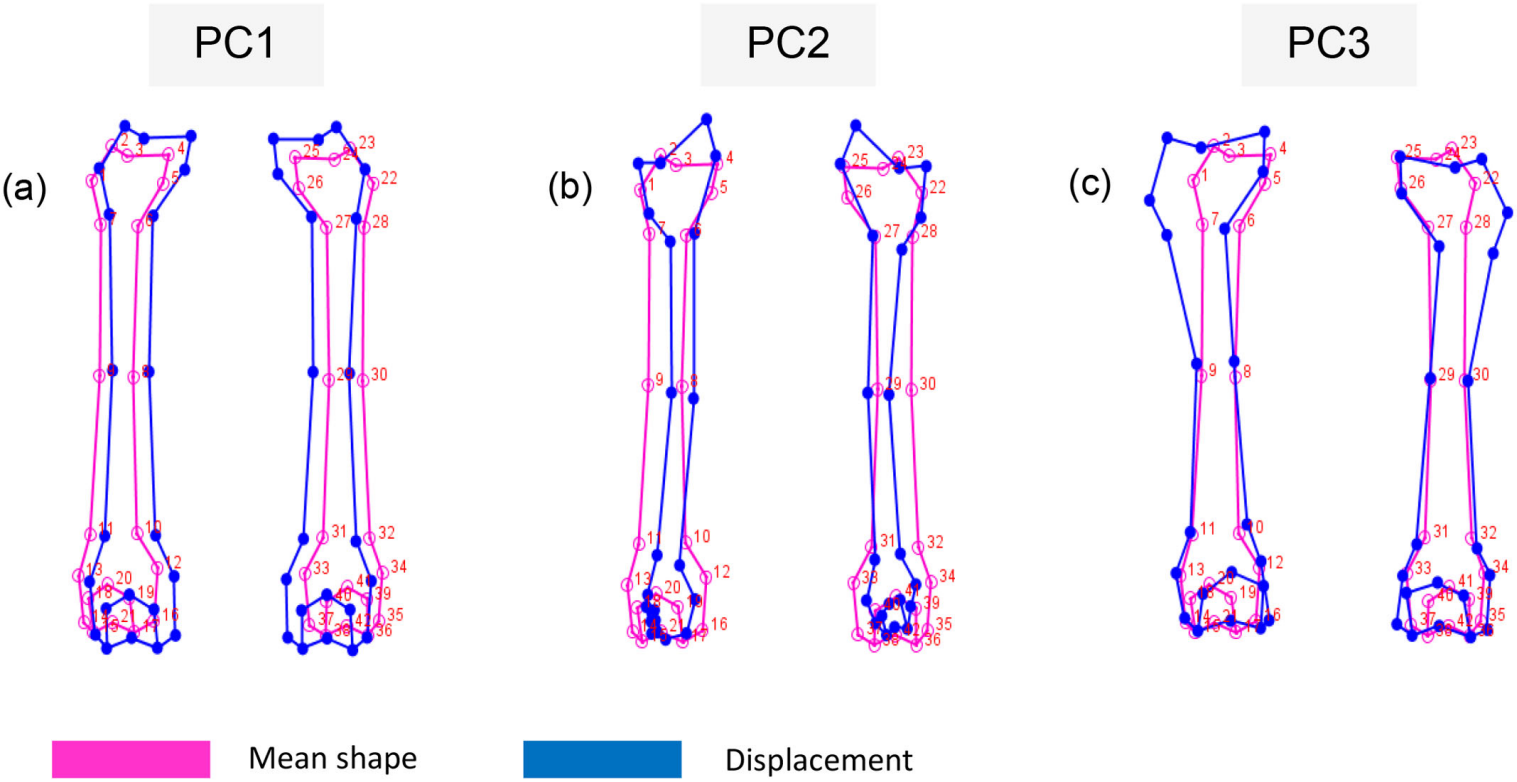
**Morphological shape changes of the *Panthera* femurs shown by the displacement from the mean shape for the first three principal components for the anterior view.** Mean shape is represented in pink and displacement is represented in blue. PC, principal component.

For particular biologically significant aspects on each femur's proximal extremities, shaft, and distal extremities, landmarks were placed, as illustrated in Fig. 1. Principal components were used as an indication of the variations within the two datasets. The statistics software used was MorphoJ v2.0 (Klingenberg, 2011), which includes an orthogonal rotation method. Principal components 1, 2 and 3 account for 93.5% (Fig. S1) of the total shape variance for the anterior view of the femurs (correlation matrix=0.75; *P*<0.0001) (Fig. 2). Principal components 1-5 account for 92.7% of the total shape variance for the posterior view of the femurs (correlation matrix=0.50; *P*<0.0001) (Fig. 2). The principal component analysis (PCA) plots for the anterior and posterior views of the *Panthera* femurs (Figs 3 and 4) show distinct clusters linking the leopard and lion species for both the anterior and posterior views. The relationships between principal component 1 (PC1) (78.8% of the total shape variance), principal component 2 (PC2) (10.9% of the total shape variance) and principal component 3 (PC3) (3.8% of the total shape variance) for the anterior view of the femurs are shown in Fig. 3. Positive PC1 scores are associated with a slightly thickened shaft as well as an overall increased size of the femur (Fig. 5A). PC1 scores on the negative end of the axis are associated with an elongated and more gracile femur. Positive PC2 scores are associated with a considerably thinner shaft as opposed to the mean femur shape and a narrowing of both the proximal and distal ends of the shaft. The femoral head can be seen progressing upwards, thereby slightly increasing the total length of the femur. There is also a reduction in the size of the distal end of the femur, including highly reduced medial and lateral epicondyles (Fig. 5B). This morphology is more related to the anterior view of the leopard femur. Negative PC2 scores most likely resemble those of a lion femur due to the thickening of the shaft, as well as the more pronounced proximal and distal ends of the femur. Positive PC3 scores are associated with an overall robust and stunted femur with robust proximal and distal ends, including broader proximal and distal ends of the shaft, enlarged epicondyles, and a greater patellar surface. An increased femoral head can also be seen progressing upward and outward (Fig. 5C). This morphology is evident in the anterior view of a lion femur. Negative PC3 scores are associated with a more slender and elongated femur shaft with reduced proximal and distal ends and smaller epicondyles. This morphology is more closely related to a leopard femur.

**Fig. 6.**
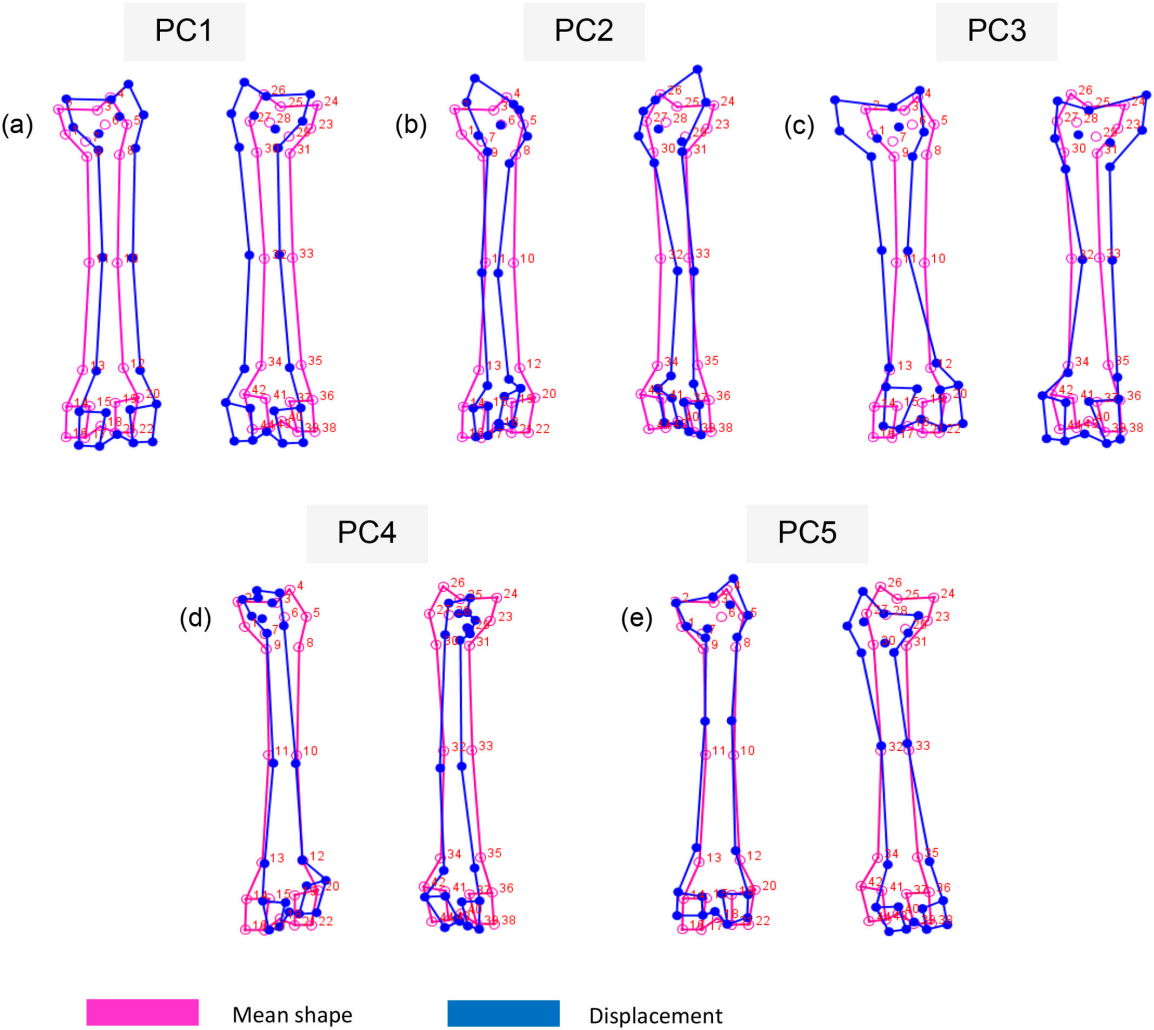
**Morphological shape changes of the *Panthera* femurs shown by the displacement from the mean shape for the first five principal components for the posterior view.** Mean shape is represented in pink and displacement is represented in blue.

The relationships between PC1 (61% of the total shape variance), PC2 (16.7% of the total shape variance), PC3 (7.6% of the total shape variance), principal component 4 (PC4) (3.9% of the total shape variance) and principal component 5 (PC5) (3.5% of the total shape variance) for the posterior view of the femurs are shown in Fig. 4. Variations shown by the PC1 scores are very similar to those of the anterior view, whereby positive PC1 scores are associated with a slightly more robust shaft (Fig. 6A), whereas negative PC1 scores are associated with a more gracile and elongated femur. Positive PC2 scores are associated with a thinner and elongated femur shaft with narrow proximal and distal ends. The size of the distal end is highly reduced with smaller medial and lateral condyles. The femoral head and neck can also be seen progressing upwards (Fig. 6B). This morphology is evident in the posterior view of the leopard femur. Negative PC2 scores are associated with a thicker shaft and more pronounced proximal and distal ends of the femur, which are more closely related to a lion femur, similar to what was shown by the PC2 scores for the anterior view. Positive PC3 scores are associated with a robust and stunted femur with a thickened shaft and broader proximal and distal ends. The condyles are enlarged, with an increased intercondyloid fossa. An increased greater trochanter can also be seen, as well as an enlarged femoral head and neck that progresses outward (Fig. 6C). This morphology is evident in the posterior view of a lion femur. Negative PC3 scores most likely resemble those of a leopard femur due to the more gracile and elongated femur shaft, reduced proximal and distal ends, smaller condyles, and decreased intercondyloid fossa. Positive PC4 scores are associated with a highly reduced proximal end of the femur, including the narrowing of the femoral head and neck region, as well as a shortened greater trochanter (Fig. 6D). PC4 scores on the negative end of the axis are more associated with a broader proximal end of the femur, including an increase in the size of the femoral head and greater trochanter. Positive PC5 scores are associated with a relatively thicker shaft with a broader distal end of the shaft. There is also a slight reduction in the size of the condyles, with the condyles having a more square-like appearance (Fig. 6E). Negative PC5 scores are associated with a slightly thinner femoral shaft, as well as condyles with a more rectangular appearance.

**Fig. 7.**
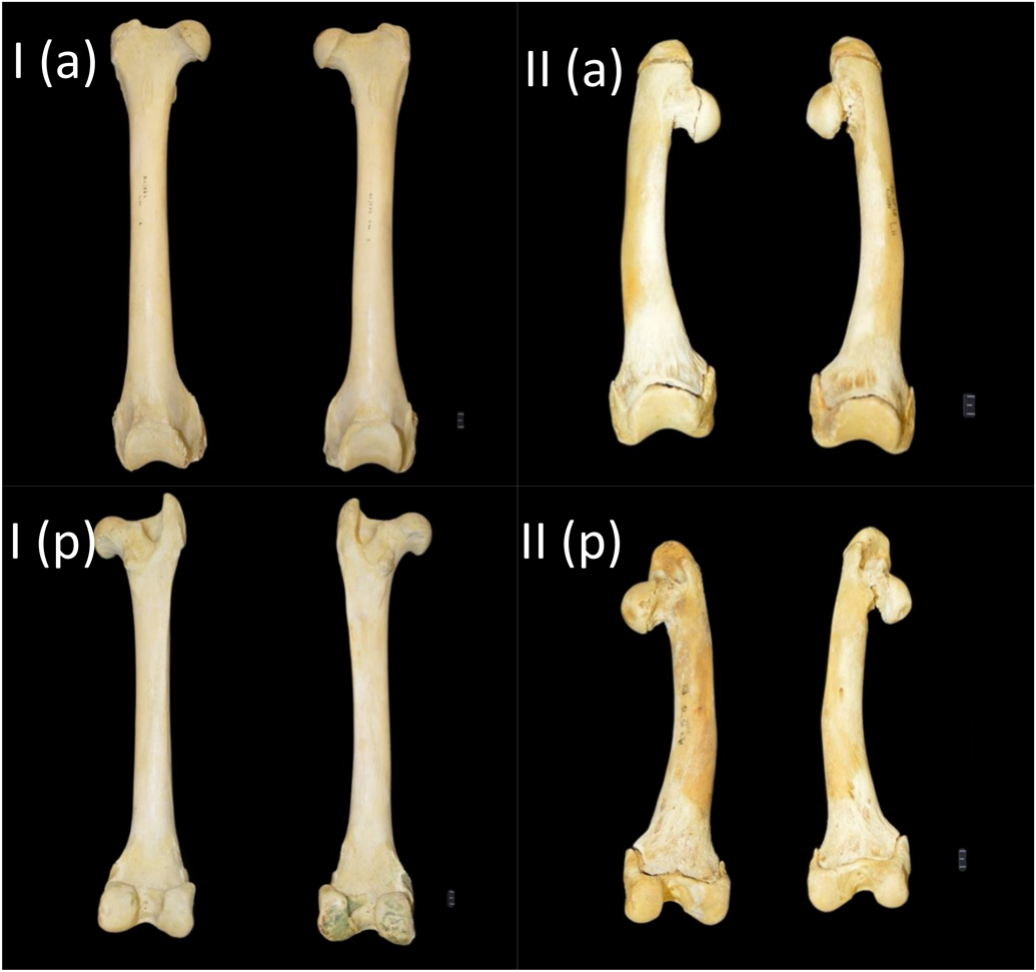
**Morphological comparisons of the femurs of a typical lion specimen and the femurs of the lion outlier in the dataset.** I, typical lion specimen; II, lion outlier in the dataset. a, anterior view; p, posterior view.

The Procrustes ANOVA regression further showed a significant difference between the mean shape of the femurs of both species, for both the anterior (*P*<0.0001; *F*=4.68) and posterior (*P*<0.0001; *F*=3.75) views. There is also one distinct outlier from the lion species with a considerably lower PC2 score, which is evident in both the anterior (Fig. 3) and posterior (Fig. 4) PCA plots. This outlier is shown in comparison to a lion specimen with PC scores that were within the lion clusters (Fig. 7). The femur of the outlier is heavily deformed and much shorter and smaller in size compared to the lion femur. The proximal end, which includes the greater trochanter and femoral head, progresses inwards, with a thicker shaft that also curves inwards. There is also a more robust distal end with an enlarged patellar surface.

## DISCUSSION

Leopards are the smallest species within the *Panthera* genus, having a body mass ranging between 30 and 80 kg (Hayward et al., 2006; Radloff and Du Toit, 2004). The hindlimbs of leopards need to be adapted to support a smaller body mass with minimal limb loading on the femurs; therefore, the morphology of these femurs does not need to be large and robust. This is evident in Fig. 5B and Fig. 6B, which show a long and slender leopard femur that is smaller in overall size, as opposed to the shorter and more robust lion femur that is larger in overall size (Fig. 5C and Fig. 6C). A lion's body mass ranges between 110 and 200 kg (Radloff and Du Toit, 2004; Owen-Smith and Mills, 2008), which drastically increases the loading forces on the hindlimbs; therefore, the shorter and more robust femur (Fig. 5C and Fig. 6C) is required to support this larger body mass to decrease bending forces. This coincides with the findings from Christiansen (1999) that larger mammals have shorter limb bones, which result in shorter limb lengths, thereby helping to reduce mechanical stress such as bending during locomotion. Shaft circumference also increases as body mass increases in order to withstand the increased limb loading (Christiansen, 1999), which is evident in our findings as the shafts of the lion femurs (Fig. 5A and Fig. 6A) are substantially thicker than those of the leopard femurs (Fig. 5B and Fig. 6B), further adding to the support of the larger body mass.

Variations in femoral shape are dependent on the respective prey size that is preferred by the predator, as there are increased biomechanical limitations that come with increasing prey sizes (Michaud et al., 2020). Studies indicate that prey size preference is strongly associated with predator body mass (Michaud et al., 2020; Owen-Smith and Mills, 2008; Gervasi et al., 2015; Tsai et al., 2016). Leopards prefer smaller prey with a body mass ranging between 10 and 40 kg, such as small herds of impala, bushbuck and duiker, all of which occur in dense habitats (Hayward et al., 2006), while lions prefer larger prey with a body mass that is almost double that of leopard prey (Radloff and Du Toit, 2004). The body mass of larger predators comes at a cost when foraging for prey as large predators expend excessive amounts of energy; therefore, this needs to be offset by capturing larger prey to increase energy intake (Michaud et al., 2020; Tsai et al., 2016). However, the capturing of larger and heavier prey results in increasing selective pressures on the hindlimbs as a more robust appendicular skeleton is required, indicating a link between bone robustness and prey preference (Michaud et al., 2020). This concurs with our findings as the predators that prey on larger animals are lions, which are characterized by more robust femurs (Fig. 5C and Fig. 6C), whereas the predators that prey on smaller animals are leopards, which are characterized by long and elongated femurs (Fig. 5B and Fig. 6B).

Leopards are solitary animals, except for mating pairs or when they are taking care of their young, meaning that they are also solitary when hunting (Spong et al., 2001; Balme et al., 2017; Miller et al., 2018). As solitary predators, they rely on habitats that provide enough cover to be able to successfully stalk their prey (Miller et al., 2018). Leopards store their prey in trees for safekeeping from other predators such as lions (Hayward et al., 2006; Miller et al., 2018). They therefore occupy habitats consisting of dense trees whereby they can increase their prey availability and hunting success. The thinner shaft coupled with the elongated leopard femur (Fig. 5B and Fig. 6B), aids with speed and flexibility during hunting in order to ou'trun their prey, or when chasing smaller prey, such as rodents, up trees. Leopards have more slender and muscular limbs that aid in generating propulsion as well as a powerful grasp that assists in arboreal locomotion (Kitchener et al., 2010; Michaud et al., 2020). The more crouched hindlimb posture allows for a lowered centre of mass, thereby increasing stability when on inclines or in arboreal habitats (Day and Jayne, 2007). Lions prefer to occupy habitats that have a high abundance of large prey (Miller et al., 2018). They generally hunt in groups to capture much larger prey, as opposed to leopards, which are solitary hunters. Therefore, lions would require more robust limbs, as seen in Fig. 5C and Fig. 6C, that are more adapted for physical strength to take down prey such as wildebeests (Michaud et al., 2020; Owen-Smith and Mills, 2008; Van Orsdol, 1984).

While both leopard and lion femurs have distinct morphological profiles, there are also variations that occur within species. The differing morphological profile shown by the lion outlier in Fig. 7 is assumed to be a result of either a birth deformity or a skeletal disease of the specimen; however, we cannot be certain, as there are no records or available details regarding this lion. A study conducted by Kutara et al. (2022) did, however, indicate a bone deformity that appears to be similar to our findings as their lion specimen also presented with short and thick long bones with a bowed shaft. It was reported that this specimen presented with a case of chondrodysplasia, which is a genetic disorder of the bone and cartilage development. Further analysis on our outlier lion bones would need to be undertaken to be able to provide a better understanding of why this femur deformation occurred, and whether this specimen has an underlying genetic disorder similar to that described by Kutara et al. (2022).

The variations in morphological profiles that were seen within clusters of species (Figs 3 and 4) could be due to the different sexes as well as the varying ages of the specimens sampled, ranging from juveniles to adolescents and adults. Male and female leopards have an average body mass of 61 and 37 kg, respectively, while male and female lions have an average body mass of 188 and 124 kg, respectively (Radloff and Du Toit, 2004). As mentioned previously, mammals with an increased body mass would need larger long bones, including femurs, which are scaled in proportion to their body mass in order to support themselves during locomotion. Therefore, males from both the leopard and lion species would have larger femurs, in contrast to those of their corresponding female species, which explains the differing femur morphological profiles shown within the clusters of species in Figs 3 and 4. In addition, this sexual size dimorphism exhibited in both leopard and lion femurs results in males being able to capture larger prey than can female individuals of the same species (Owen-Smith and Mills, 2008). This is evident in a study by Radloff and Du Toit (2004), whereby males from both leopard and lion species captured prey with a larger mean body mass of 34.2 and 399 kg, respectively, as opposed to females from both leopard and lion species, which captured prey with a much smaller mean body mass of 25.2 and 126 kg, respectively.

This study aimed to determine the extent to which the hindlimb morphology differs between *Panthera* species, primarily focusing on the difference in femur morphology between the *Panthera pardus* (i.e. leopard) and *Panthera leo* (i.e. lion). It was found that the leopard femurs had a more gracile and elongated femur morphology that assists in the support of a smaller body mass and provides flexibility for both cursorial and arboreal locomotion when hunting. The lion femurs were found to have a more robust and stunted femur morphology that assists in the support of a larger body mass and aid in cursorial locomotion. Variations in femur morphology were also found within species, which can be concluded as a result of varying age and sexual size dimorphism. The morphology ultimately verified that femur morphology differs between *Panthera* species in accordance with their mechanical demands during locomotion. It can also be used as an indication of felid locomotion and possibly to infer hunting success.

## MATERIALS AND METHODS

This study was conducted using two-dimensional landmark-based geometric morphometric methodology, adapted from Webster and Sheets (2010), in order to reduce the dimensionality of the datasets and to allow for the geometric information to be easily interpreted (Slice, 2007). A set of homologous landmarks consisting of Cartesian coordinates was used to describe the bone morphology of *Panthera* hindlimbs. The *Panthera* species that were used in this research included the *Panthera pardus* (leopard) and the *Panthera leo* (lion). Morphological characteristics of the anterior and posterior views of the femur were analysed for both species in order to determine the shape differences between the species.

### Image capturing

Digital images of the pairs of femurs were taken in the anterior and posterior views of museum specimens that were obtained from the Iziko South African Museum (33.92870°S, 18.41101°E) in Cape Town, South Africa and from the Ditsong National Museum of Natural History (25.75306°S, 28.18712°E) in Pretoria, South Africa. A total of 27 museum specimens were used throughout this study, including 21 pairs of leopard femurs and six pairs of lion femurs. The image capturing setup was kept constant to reduce error and minimize distortion by the camera. The following factors were kept constant: the camera (D3500, Nikon, Japan), distance from the bone (40 cm), unidirectional lighting, and background. Images were also edited using PhotoScissors v8.3 (Teorex, USA) in order to remove unwanted objects (i.e. noise), background, adjust the contrast or brightness, and flip images to ensure they were all in the same orientation.

### Landmarking

The software used for landmarking was ImageJ v1.53s (Schneider et al., 2012). Each of the edited images was imported into ImageJ and landmarked separately to quantify and analyse the morphological differences between the femurs of both species. Excel 2010 (Microsoft Corporation, USA) spreadsheets were used to record the *x*-*y* coordinates of each landmark, with separate spreadsheets being used for the anterior and posterior views. A subset of 42 landmarks were placed on the anterior view of the pairs of femurs and 44 landmarks on the posterior view (Table 1). Landmarks were positioned, as shown in Fig. 1, for specific features with biological significance on the proximal extremities, shaft, and the distal extremities of each femur. The proximal extremities included the femoral head, neck, greater trochanter, trochanteric fossa, and the lesser trochanter (Kappelman, 1988; Serrat et al., 2007; Michaud et al., 2020). The distal extremities included the medial epicondyles, medial condyles, intercondyloid fossa, lateral epicondyles, lateral condyles and patellar surface (Kappelman, 1988; Serrat et al., 2007; Michaud et al., 2020). Landmarks were positioned in the same order for each image, with each image having the same number of landmarks. Landmarks were added separately for the anterior view and the posterior view. The same scale factor of 1 cm was set for all images, and an ID was set for each image to maintain consistency. The landmark spreadsheets were saved as tab-delimited text files.

**Table 1.**
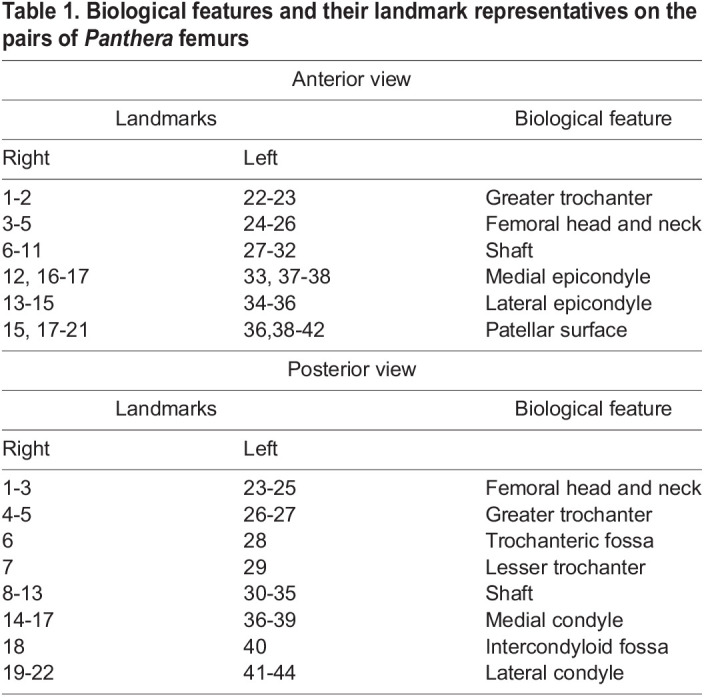
Biological features and their landmark representatives on the pairs of *Panthera* femurs

### Analyses

The anterior and posterior text files comprising of the landmark coordinates were imported separately into MorphoJ v2.0 (Klingenberg, 2011). This is a software package used for geometric morphometrics, where the analyses were carried out separately for each view of the femurs. A Procrustes fit was generated to analyse the distribution of the *Panthera* femur morphology to determine the best fit between the landmarked femurs. A Procrustes distance was also calculated to check the degree of similarity of the shapes. A wireframe was created, and classifiers were added from the datasets (i.e. species). A covariance matrix for the data was then created using the generated Procrustes coordinates. PCA was used to reduce and simplify the dimensionality of the dataset, while still maintaining the patterns and trends in the data. PCA plots were generated for the landmarked femurs to reveal any distinct morphological clusters in both the anterior and posterior views of the femurs. A Procrustes ANOVA regression model was then used to test the difference in mean shape of the femurs between the different *Panthera* species, for both the anterior and posterior views separately.

## Supplementary Material

10.1242/biolopen.061823_sup1 Supplementary information
