# Supplementary material for: The relationship between locomotion and hindlimb morphology in the leopard (Panthera pardus) using a geometric morphometric approach
Source: Biol Open. 2024 Dec 24;13(12):bio061823. doi: 10.1242/bio.061823 (PMC11708771; doi:10.1242/bio.061823)
Supplement: Supplementary information [file biolopen-13-061823-s1.pdf]

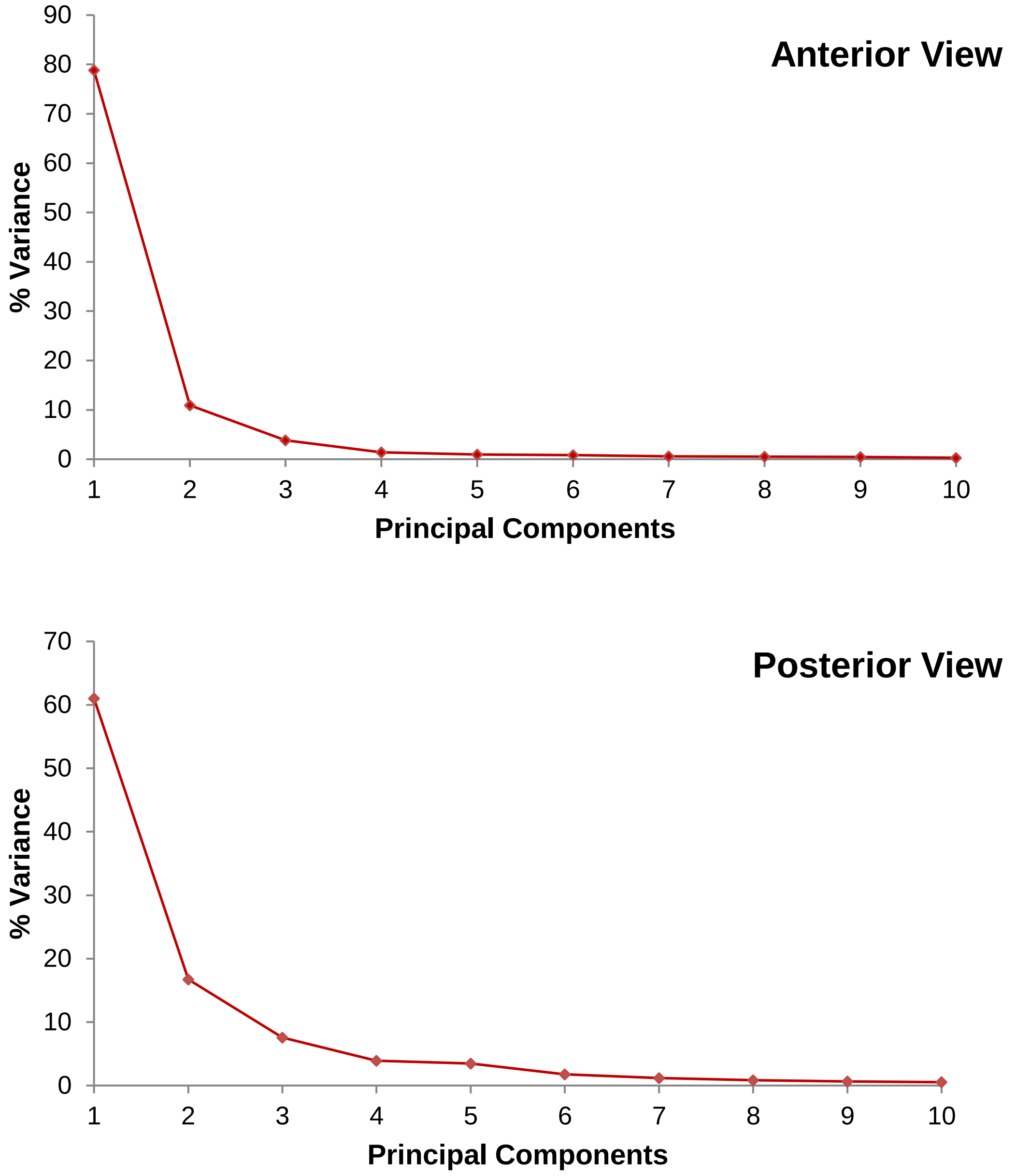

Fig. S1. Scree plot representing the percentage variance of the first ten principal components for both the anterior and posterior views of the Panthera femurs.

Biology Open • Supplementary information
